# Incisional surface quality of electron-beam irradiated cornea-extracted lenticule for stromal keratophakia: high nJ-energy vs. low nJ-energy femtosecond laser

**DOI:** 10.3389/fmed.2023.1289528

**Published:** 2023-12-15

**Authors:** Jian S. Chan, Evelina Han, Chris H. L. Lim, Arthur C. Kurz, Jeremy Shuman, Yu-Chi Liu, Andri K. Riau, Jodhbir S. Mehta

**Affiliations:** ^1^School of Clinical Medicine, University of New South Wales, Sydney, NSW, Australia; ^2^Department of Ophthalmology, National University Health System, Singapore, Singapore; ^3^Tissue Engineering and Cell Therapy Group, Singapore Eye Research Institute, Singapore, Singapore; ^4^Yong Loo Lin School of Medicine, National University of Singapore, Singapore, Singapore; ^5^Lions World Vision Institute, Tampa, FL, United States; ^6^Ophthalmology and Visual Sciences Academic Clinical Programme, Duke-NUS Medical School, Singapore, Singapore; ^7^Singapore National Eye Centre, Singapore, Singapore

**Keywords:** femtosecond laser, electron-beam sterilization, cornea, refractive lenticule, surface, collagen, SMILE, CLEAR

## Abstract

**Introduction:**

Corneal lenticules can be utilized as an additive material for stromal keratophakia. However, following extraction, they must be reimplanted almost immediately or cryopreserved in lenticule banks. Electron-beam (E-beam) irradiated corneas permit room-temperature storage for up to 2 years, enabling keratophakia to be performed on demand. This study aims to compare the performance of high nano Joule (nJ)-energy (VisuMax) and low nJ-energy (FEMTO LDV) femtosecond laser systems on the thickness consistency and surface quality and collagen morphology of lenticules produced from fresh and E-beamed corneas.

**Methods:**

A total of 24 lenticules with −6.00 dioptre power were cut in fresh human donor corneas and E-beamed corneas with VisuMax and FEMTO LDV. Before extraction, the thickness of the lenticules was measured with anterior segment-optical coherence tomography (AS-OCT). The incisional surface roughness of extracted lenticules was analyzed using atomic force microscopy (AFM) and scanning electron microscopy (SEM). Multiphoton microscopy was then used to assess the surface collagen morphometry.

**Results:**

The E-beamed lenticules that were cut using FEMTO LDV were significantly thicker than the fresh specimens as opposed to those created with VisuMax, which had a similar thickness as the fresh lenticules. On the vertex, they were ∼11% thicker than the fresh lenticules. The surface roughness (R_q_) of E-beamed lenticules incised with FEMTO LDV did not differ significantly from the fresh lenticules. This contrasted with the VisuMax-fashioned lenticules, which showed notably smoother surfaces (∼36 and ∼20% lower R_q_ on anterior and posterior surfaces, respectively) on the E-beamed than the fresh lenticules. The FEMTO LDV induced less cumulative changes to the collagen morphology on the surfaces of both fresh and E-beamed lenticules than the VisuMax.

**Conclusion:**

It has been previously demonstrated that the low nJ-energy FEMTO LDV produced a smoother cutting surface compared to high nJ-energy VisuMax in fresh lenticules. Here, we showed that this effect was also seen in the E-beamed lenticules. In addition, lower laser energy conferred fewer changes to the lenticular surface collagen morphology. The smaller disparity in surface cutting quality and collagen disturbances on the E-beamed lenticules could be beneficial for the early visual recovery of patients who undergo stromal keratophakia.

## 1 Introduction

The continuing development of femtosecond lasers has enabled minimally invasive surgical techniques, such as lenticule extraction (1, 2). The first commercially available femtosecond laser system capable of this was the VisuMax developed by Carl Zeiss Meditec (Jena, Germany), which trademarked their ReLEx procedure as Small Incision Lenticule Extraction (SMILE) (3, 4). More recently Ziemer Ophthalmic Systems (Port, Switzerland), who developed the FEMTO LDV, introduced a similar procedure referred to as cornea Lenticule Extraction for Advanced Refractive-Correction (CLEAR) (5, 6). Although the lasers have different technical specifications and employ different photodisruptive processes—VisuMax employs high nano Joule (nJ)-energy and low-frequency pulses, while FEMTO LDV employs low nJ-energy and high-frequency pulses (Table 1), both lenticule extraction procedures involve precise laser firings that photo-disrupt the stromal material creating a posterior and then anterior lenticular surface. The result is a precisely sculpted lenticule within the corneal stroma. The lenticule is retrieved through a small incision and normally disposed of as medical waste (7, 8). We have previously shown over a decade ago, in our proof-of-concept studies, of lenticule reimplantation, as a modern iteration of stromal keratophakia (9, 10). The tissue addition procedure was subsequently clinically implemented in the treatment of several conditions, including keratoconus, hyperopia, presbyopia, and corneal perforations (11–14). These lenticules can be sourced from living allogeneic donors, who had undergone ReLEx to correct myopia (15, 16), or cryopreserved autologous and allogeneic lenticules, retrieved from a lenticule bank (17). However, lenticules may also be created from cadaveric donor corneal tissues (18), or OptiGraft^®^ (Lions World Vision Institute, Tampa, FL, USA), commercially available electron-beam (E-beam) irradiated corneas (19).

**TABLE 1:** Comparison of technical specifications between VisuMax and FEMTO LDV femtosecond laser systems.

	VisuMax*	FEMTO LDV
Emission source	Fiber amplifier	Oscillator
Wavelength	1,043 nm	1,020–1,060 nm
Pulse width	220 to 580 fs	250–350 fs
Spot size	∼1 μm	< 1 μm
Repetition rate	500 kHz	> 5,000 kHz
Pulse energy	110–150 nJ	<100 nJ
Operation speed (at 9.5 mm)	Between 20 and 60 seconds	<40 Seconds
Mobility	Fixed	Portable
Environmental requirements	Room temperature/humidity can vary	Room temperature/humidity can vary
Docking method	Sliding patient under machine on fixed bed	Floating mobile handpiece
Contact glass	Curve	Flat
Interoperative OCT	No	Yes
Automatic pupil detection	No	Yes
Pupil center offsetting	No	Yes
Real-time video recording	Yes	No

*VisuMax 800 kHz is now commercially available but VisuMax 500 kHz was used in the current study.

E-beam irradiation is an emerging sterilization method. The appeal of using E-beam irradiated corneas is that they can be stored at room temperature for up to 2 years (20). Unlike frozen and glycerine-preserved corneas, these corneas can be used without reconstitution. They also remain transparent and do not discolor over time compared to gamma rays irradiated corneas (21, 22). Following E-beam treatment, the corneas are stored in a hydrated state in plant-derived recombinant human serum albumin (rHSA). The rHSA is selected because it does not pose immunogenicity risks and risks of transmission of blood-borne infectious diseases associated with human-derived serum products (23). Furthermore, E-beam irradiation reduces the risk of infections caused by microbial pathogens (24, 25). Hence, the availability of E-beam irradiated corneas has the potential to reduce tissue wastage at eye banks and allow the use of transplant-grade donor corneas for higher-priority cases.

Historically, stromal keratophakia has been an abandoned procedure due to postoperative complications and suboptimal outcomes. Many of these issues were due to technical limitations at the time when it was first described by José Ignacio Barraquer in the 1960s (7). The refractive lenticules were cut freehand or using a manual microtome out of frozen donor corneas and as a result had roughened surfaces and inaccurate thickness (26–28). Upon thawing and implantation of the lenticules, it predisposed patients to several complications, including corneal oedema and an increase in astigmatism (26–28).

Incisional surface quality is important for early visual recovery following corneal refractive surgeries (29–31). For example, a prospective non-randomized study conducted on patients undergoing SMILE found that surface interface roughness was inversely associated with corrected distance visual acuity (CDVA) (31). This effect was observed only on the one-day post-operative follow-up as the surface irregularities may reduce due to stromal collagen remodeling postoperatively. In addition, Kamiya et al. (29) demonstrated that surface irregularity at the lamellar interface has been shown to increase backscattering of light and was strongly associated with poorer CDVA.

To the best of our knowledge, all reported clinical cases of stromal keratophakia have only been performed with lenticules extracted with SMILE. More recently, other than CLEAR, several other lenticule extraction procedures that use low nJ-energy laser systems have become available, such as SmartSight (Schwind, Kleinostheim, Germany) and small incision lenticule keratomileusis (SILK; Johnson & Johnson Vision, Irvine, CA, USA) (32, 33). Hence, we sought to compare the differences in performance between two of these lasers, the VisuMax and FEMTO LDV with respect to lenticule extraction. In response to the implications of surface quality for clinical outcomes, we assessed the incisional surface quality of the lenticules excised with scanning electron microscopy (SEM) and quantitatively examined with atomic force microscopy (AFM). In addition, the collagen morphometry at the lenticular surfaces was evaluated using multi-photon microscopy. We also assessed the femtosecond laser cutting consistency through anterior segment-optical coherence tomography (AS-OCT) 3D and line scans.

## 2 Materials and methods

### 2.1 Donor cornea materials and experimental design

**FIGURE 1:**
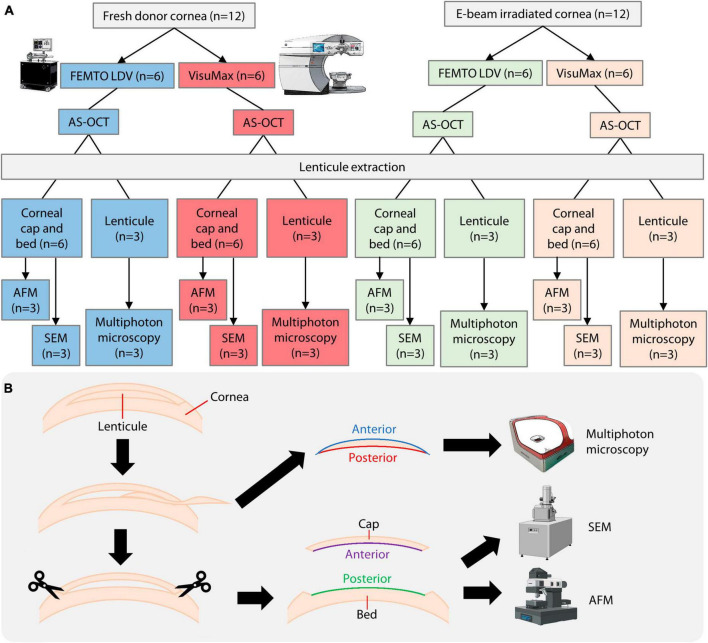
Overview of experimental procedures. **(A)** Breakdown of donor material usage and methods employed. **(B)** To fully utilize the donor corneas, corneal caps and stromal beds were used as surrogate surfaces of lenticules for AFM and SEM, while the lenticules were used for multiphoton microscopy. E-beam, electron-beam. AS-OCT, anterior segment optical coherence tomography; AFM, atomic force microscopy; SEM, scanning electron microscopy. A part of the figure was created with Biorender.com (agreement no. LU25RI9WXL).

A comparative experiment was performed to study the outcomes of the VisuMax and FEMTO LDV femtosecond laser systems in cutting lenticules from donated human corneal specimens (fresh group; *n* = 12; Lions World Vision Institute) and E-beam irradiated corneas (E-beam group; *n* = 12) (Figure 1A). Ethics approval and informed consent collection were carried out by the US eye bank and were not required to be repeated in Singapore. The fresh corneas were shipped in Optisol-GS media (Bausch+Lomb, Bridgewater, NJ, USA) and underwent laser procedures within 1 day of receiving. The E-beam irradiated corneas were kindly provided by the Lions World Vision Institute. The irradiation was performed by SteriTek (Fremont, CA, USA) using two 10 MeV, 20 kW linear electron accelerators. Each cornea was stored in a sealed vial containing 20% rHSA (InVitria, Aurora, CO, USA). A box holding 50 vials of corneas was rolled through the electron accelerator one time with an external dosage of 19–23 kGy, which corresponded to an internal dosage of 17–25 kGy. The E-beam irradiated corneas were stored for 1 year before the subsequent experiments were conducted.

### 2.2 Refractive lenticule creation

Fresh and E-beam treated corneas were rinsed and equilibrated in 1X phosphate-buffered saline (PBS; 1st BASE, Singapore, Singapore) for 1 h before CLEAR or SMILE procedures. CLEAR and SMILE were performed on the corneal specimens using the FEMTO LDV Z8 and VisuMax 500 kHz femtosecond laser systems, respectively. The laser parameters of both femtosecond laser systems were set to cut the lenticules with the following profile: −6.00D myopic correction, 110 μm cap thickness, 7.5 mm cap diameter, and 6.5 mm lenticular diameter. An energy level of 140 nJ was set to cut the anterior and posterior surfaces of SMILE lenticules. For CLEAR lenticules, an energy setting of 38 and 40% was used to cut anterior and posterior surfaces, respectively. After the laser firing sequence, AS-OCT was performed to measure the lenticule thickness *in situ* (further described in the section below). Under a surgical microscope, the lenticules were separated from the stromal cap and bed surfaces using a lamellar dissector (ASICO, Westmont, IL, USA) and carefully retrieved through the small incision using Tan DSAEK forceps (ASICO).

### 2.3 Laser cutting consistency

To assess the lenticular thickness and cavitation bubble formation within the corneas, line scans and 3D scans were obtained using an RTVue AS-OCT (OptoVue, Inc., Fremont, CA, USA), respectively. Lenticular thickness measurements were taken from the anterior cap to the residual stromal bed borders at the center (0.0 mm), ± 1.0, ± 2.0, and ± 3.0 mm positions of the lenticule. Position 0 was equivalent to the central axis of the cornea specimens.

### 2.4 Incisional surface quality

Following lenticule extraction, the corneal cap and stromal bed surfaces were subjected to SEM and AFM and used as surrogate surfaces of the lenticules (Figure 1B). The corneal cap and stromal bed represented the anterior and posterior surfaces of the lenticules. The lenticules were used for multiphoton microscopy, which will be described in the following section. Post-lenticule extraction fresh and E-beamed corneas were fixed in 2.5% glutaraldehyde (Sigma-Aldrich, St Louis, MO, USA) at 4°C overnight, followed by post-fixation in 1% osmium tetroxide for 2 hours at room temperature. After rinsing, the samples underwent a dehydration procedure in a series of increasing concentrations of ethanol for 10 min each, from 25 to 100% ethanol (Merck Millipore, Burlington, MA, USA), followed by critical point drying in a Leica EM CPD300 system (Leica Biosystems, Wetzlar, Germany). The stromal caps were snipped off with surgical scissors and mounted stroma side up on mica sheets (Electron Microscopy Science, Hatfield, PA, USA). The residual stromal beds were mounted stroma side up on separate mica sheets. Dimension Icon AFM (Bruker, Billerica, MA, USA) was used to scan tissue surfaces in tapping mode. Images were captured in 256 × 256 in resolution and at 1 Hz frequency. Six random areas in the central 3 mm and the periphery of each sample were selected for further analysis of surface roughness (R_q_ and R_z_). The remaining stromal cap and bed tissues were mounted onto SEM sample stubs and coated with a 15 nm layer of gold using a Leica EM ACE200 sputter coater (Leica Microsystems, Wetzlar, Germany). A Quanta 650 FEG scanning electron microscope (SEM) (FEI Company, Hillsboro, OR, USA) was then used to scan the surfaces of the corneal cap and stromal bed tissues.

### 2.5 Collagen morphometry of incisional surface

Lenticules extracted from the fresh and E-beam-treated corneas were fixed with 4% paraformaldehyde (Sigma-Aldrich) and embedded in paraffin blocks. The sample was mounted on a HistoCore microtome (Leica Biosystems) and sectioned into 4 μm slices. These unstained sections were then scanned using a Genesis 200 multiphoton imaging platform (HistoIndex Pte Ltd., Singapore). Two-photon excitation (TPE) and second harmonic generation (SHG) signals were captured following laser excitation of the samples at 780 nm. The SHG signal was captured using photomultiplier tubes set at 390 nm wavelength and using a 450DCLP dichroic mirror, the TPE signal was segregated from the SHG signal at 550 nm wavelength. Images of lenticules were taken at 20× magnification with a dimension of 200 μm^2^ × 200 μm^2^ and generated at 512 × 512-pixel resolution. Multiple adjacent image tiles were captured and automatically stitched to encompass the entire lenticule on each slide. Collagen profiles of the entire anterior and posterior surfaces, assessed from the surface down to 5 μm depth, and the bulk stroma of the lenticules were then analyzed with FibroIndex software (HistoIndex Pte Ltd.).

### 2.6 Statistical analysis

All values were reported as mean ± standard error of the mean. The statistical comparisons between two experimental groups were carried out with the Mann–Whitney U test and between multiple groups were conducted with Welch’s ANOVA followed by Games-Howell *post-hoc* test on SPSS version 17.0 (IBM, Armonk, NY, USA). Comparisons of R_q_ and R_z_ in the central and peripheral surfaces within the same samples were carried out with Paired Samples *T*-Test. A *p*-value lower than 0.05 was considered to be statistically significant.

## 3 Results

### 3.1 Laser cutting consistency and coalescence of cavitation bubbles

**FIGURE 2:**
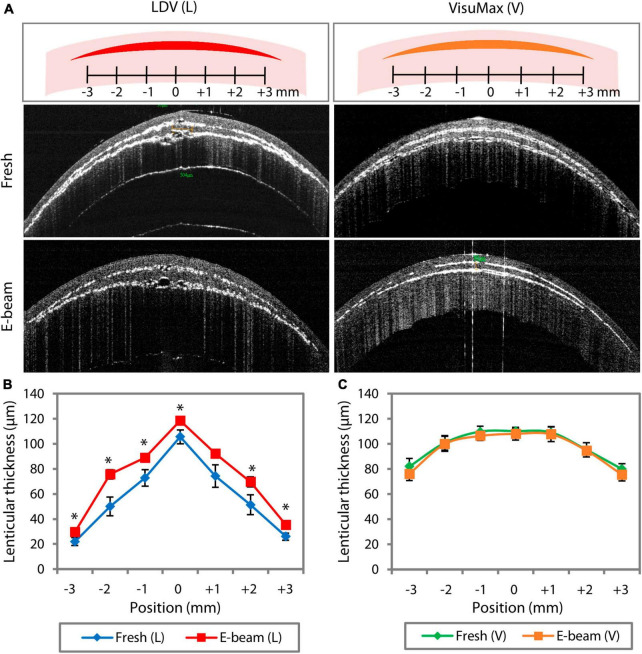
FEMTO LDV (L)- and VisuMax (V)-assisted lenticule cutting consistency in fresh and E-beam irradiated corneas. The lenticular thickness measurements were taken *in situ* before the lenticule extraction. **(A)** Representative AS-OCT line scan images showed the presence of myopic lenticules within the fresh and E-beam-treated corneas. **(B)** Measured at the same 7 different positions within the corneas, the thickness of E-beamed lenticules was significantly higher than the fresh counterparts when cut with the FEMTO LDV. **(C)** In contrast, the thickness of lenticules appeared to be similar in both types of corneas when incised with the VisuMax. Error bars indicate the standard error of the means. Position 0 marks the central axis of the cornea specimen. *p < 0.05.

In the AS-OCT line scans, crescent-shaped fresh and E-beamed lenticules were observed in all experimental groups, with the VisuMax-incised lenticules showing a more gradual tapering from the center to the periphery (Figure 2A). Other notable differences included the laser cutting consistency and the tendency of coalesced cavitation bubble formation. The E-beamed lenticules cut with the FEMTO LDV were significantly thicker than the fresh lenticules (Figure 2B and Supplementary Table 1). On the vertex, they were approximately 11% thicker than the fresh lenticules. In contrast, the thickness of lenticules created by VisuMax remained consistent in the fresh and E-beam-irradiated corneas (Figure 2C and Supplementary Table 1). In the AS-OCT 3D scans, no coalescence of cavitation bubbles was noticed on any lenticule-stroma interface of both fresh and E-beam lenticules after the CLEAR procedure (Supplementary Videos 1, 2). However, the coalescence of cavitation bubbles was not observed on any side of the fresh SMILE-derived lenticules (Supplementary Video 3), but could be frequently distinguished in the posterior and periphery region of the E-beam SMILE-derived lenticules (Supplementary Video 4)—-an observation consistent with our published work (19).

### 3.2 Qualitative and quantitative incisional surface morphology

**FIGURE 3:**
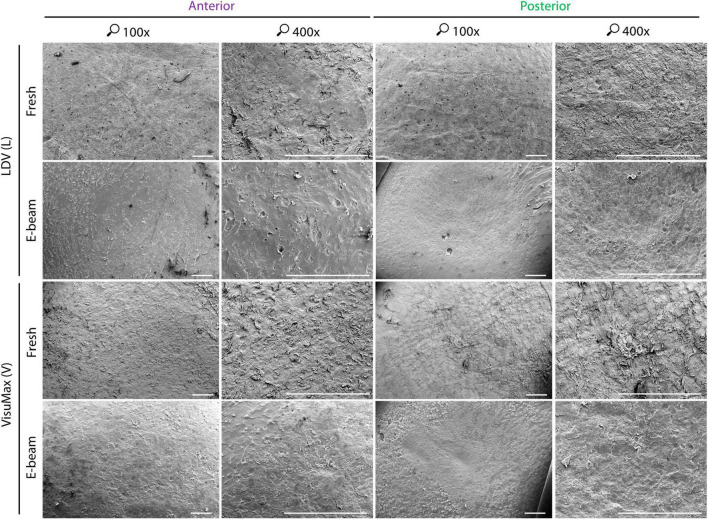
Qualitative surface morphology of corneal caps and stromal beds assessed with scanning electron microscopy. To fully utilize the donor corneas, corneal caps and stromal beds were used as surrogate anterior and posterior surfaces of lenticules, respectively. The anterior and posterior surfaces of the FEMTO LDV (L)-cut fresh lenticules appeared smooth. A similar observation of roughness could be seen on the FEMTO LDV-incised E-beamed lenticule on their anterior and posterior surfaces. Severed tissue bridges, appearing as bundles of collagen fibers, on the lenticular surfaces were not as abundant as the VisuMax (V)-lasered fresh lenticule, which had consistent rough texture throughout the anterior and posterior surfaces. The E-beamed lenticule created with VisuMax, however, appeared smoother than the fresh lenticule resembling the FEMTO LDV-cut lenticules. Images were taken at 100× and 400× magnification. Scale bars = 500 μm.

Qualitatively, the anterior and posterior surfaces of fresh and E-beamed lenticules in the FEMTO LDV group appeared to have a smooth texture in the center and rougher morphology in the periphery (Figure 3). The surface roughness on the lenticular surface was typically attributed to tissue bridges—-bundles of collagen fibers not affected by laser-induced cavitation bubbles (34, 35). In comparison to the FEMTO LDV-lasered fresh lenticular surfaces, the VisuMax-cut anterior surface appeared rougher with abundant tissue bridges. The posterior surface seemed to have an even rougher texture and regularly featured thicker clumps of collagen fibers. The E-beamed SMILE lenticules, however, had a smoother surface than their fresh counterparts.

**FIGURE 4:**
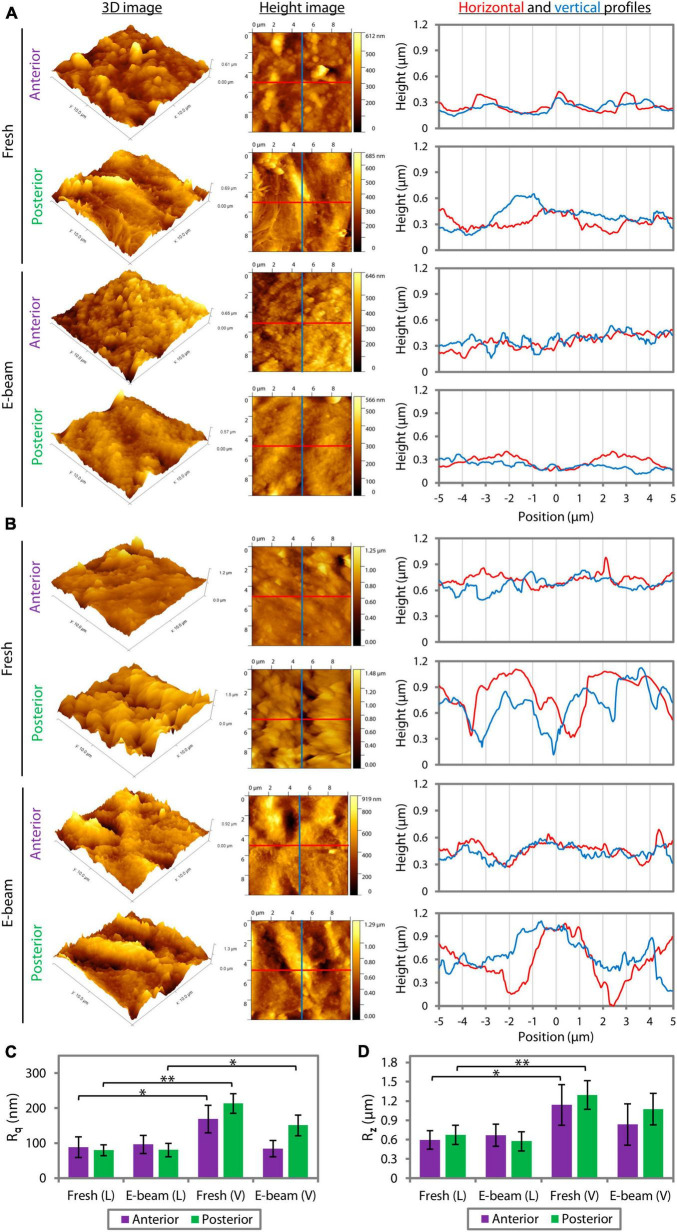
Quantitative surface roughness of corneal caps and stromal beds assessed with atomic force microscopy. The 3D and height images and lateral height profiles (derived from the height images) captured the differences in the lenticular surface roughness incised with FEMTO LDV **(A)** and VisuMax **(B)**. **(C)** The root mean square of roughness (R_q_) of the anterior and posterior surfaces of the FEMTO LDV (L)-cut lenticules, extracted from either fresh or E-beam irradiated corneas, was similar. In contrast, the fresh lenticules lasered with VisuMax (V) had relatively rougher surfaces than the E-beam counterparts. **(D)** Average maximum roughness (R_z_) followed the same pattern of outcomes as the R_q_. Error bars indicate the standard error of the means. *p < 0.05. **p < 0.001.

Atomic force microscopy (AFM) revealed that the roughness profiles, either the root mean square (R_q_) (Supplementary Figure 1A) or average maximum roughness (R_z_) (Supplementary Figure 1B), of the center and periphery of lenticules that had the same pre-treatment (fresh or E-beam irradiated) and were cut with the same femtosecond laser system (FEMTO LDV or VisuMax) did not differ significantly (Supplementary Table 2). Henceforth, the presented R_q_ and R_z_ were the average values taken at the center and periphery combined. AFM showed that CLEAR-derived lenticules (Figure 4A) had smoother surfaces compared to SMILE-derived lenticules (Figure 4B). The R_q_ of the fresh and E-beamed lenticular surfaces was significantly lower in the FEMTO LDV than VisuMax groups, except the anterior surfaces of E-beamed samples (Figure 4C and Supplementary Table 3). The R_q_ of the anterior and posterior surfaces of the FEMTO LDV-cut lenticules was similar whether the lenticules were extracted from the fresh or E-beam treated corneas (Figure 4C and Supplementary Table 3). This contrasted with the VisuMax lenticules, where the fresh lenticules had markedly rougher anterior (*p* = 0.001) and posterior surfaces (*p* = 0.005) than the E-beam lenticules (Figure 4C and Supplementary Table 3). In both types of lenticules, the posterior aspect appeared to have rougher topography than the anterior aspect (Figures 4B, C and Supplementary Table 3). Correspondingly, the R_z_ of the lenticular surfaces in either FEMTO LDV or VisuMax groups followed the same pattern of outcomes as the R_q_ (Figure 4D and Supplementary Table 3).

### 3.3 Collagen morphometry at incisional surfaces

**FIGURE 5:**
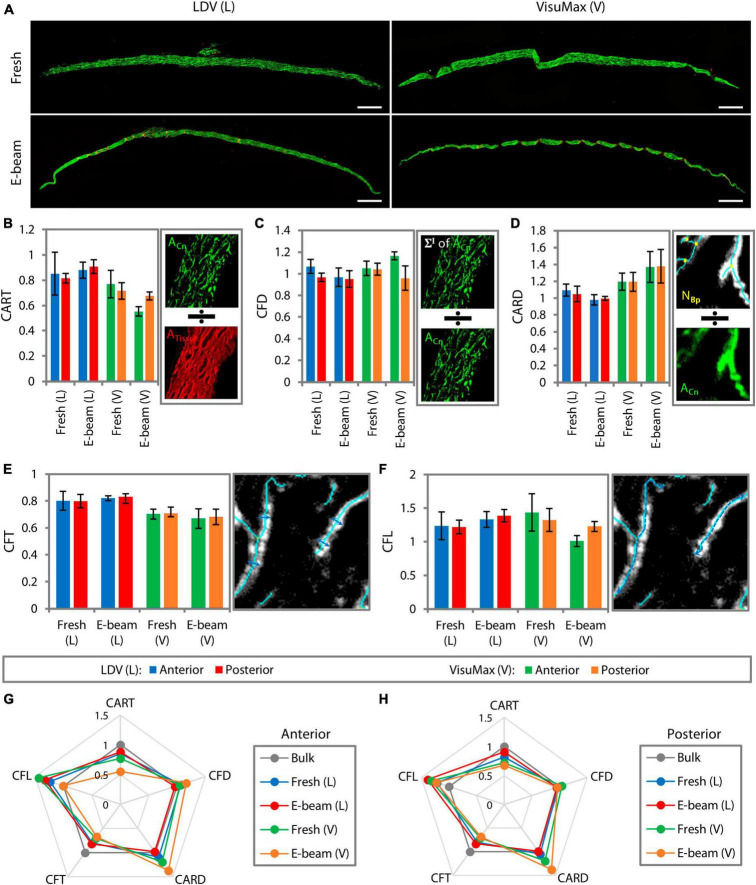
Surface collagen morphometry of the lenticules resolved with multiphoton microscopy. **(A)** Images of lenticules were taken with a dimension of 200 μm^2^ × 200 μm^2^. Multiple adjacent image tiles were stitched to encompass the entire lenticule. Scale bars = 200 μm. Due to some differences in the collagen morphometry induced by E-beam irradiation, measurement parameters of the surfaces of each lenticule were normalized to those of its respective bulk stroma. Collagen morphometry, such as collagen area ratio in tissue (CART) **(B)**, collagen fiber density (CFD) **(C)**, collagen area reticulation density (CARD) **(D)**, collagen fiber thickness (CFT) **(E)**, and collagen fiber length (CFL) **(F)**, was generated by the imaging system. Depictions of how the system generated each measurement parameter are presented on the right of each bar graph. Radar maps summarizing the collagen morphometry of the anterior **(G)** and posterior surfaces **(H)** relative to that of the bulk stroma. The maps revealed that the surface collagen morphometry was most affected in the VisuMax-incised lenticules, particularly the E-beam lenticules.

Representative multiphoton microscopy stitched images showed marginal differences in the collagen intensity of fresh and E-beamed lenticules (Figure 5A). Each measurement parameter of the surface was normalized to that of the bulk stroma. The collagen morphology was found to be similar between fresh and E-beamed bulk stroma (Supplementary Table 4). The measurement outcome would therefore represent a differential ratio to the bulk stroma. Collagen area ratio in tissue (CART), the relative amount of collagen over the total tissue area, appeared to be reduced in the anterior and posterior surfaces of both fresh and E-beamed lenticules in the FEMTO LDV group (Figure 5B and Supplementary Table 4). Incisions with VisuMax seemed to have an even more pronounced effect on the CART of the lenticular surfaces, particularly in the E-beam samples (44.84 ± 3.80% change in the anterior surface and 32.40 ± 3.00% in the posterior surface) (Figure 5B and Supplementary Table 5). Both femtosecond laser systems altered the collagen fiber density (CFD), which was obtained by the sum of each pixel within the collagen area multiplied by its corresponding intensity over the area of collagen, to a rather similar level in any surface of either fresh and E-beamed lenticules (Figure 5C and Supplementary Table 5). The highest alterations in CFD were noted in the E-beam SMILE-derived lenticules, where the anterior surface’s CFD had a 16.49 ± 3.68% change and the posterior surface’s CFD had a 16.31 ± 1.10% change. The collagen area reticulation density (CARD), the ratio of the number of branch points relative to the area of collagen, was similar in the surfaces of fresh and E-beamed lenticules created with FEMTO LDV (Figure 5D and Supplementary Table 5). There was a further increase in CARD in the surfaces of fresh and E-beam lenticules lasered with the VisuMax (Figure 5D and Supplementary Table 5). Similar to the first two measurement parameters, the largest change was found in the E-beamed lenticules after SMILE.

The collagen fiber thickness (CFT) was equally reduced in the anterior and posterior surfaces of fresh and E-beamed lenticules following incisions with FEMTO LDV (Figure 5E and Supplementary Table 5). The CFT was reduced further in the surfaces of both lenticules in the VisuMax group (Figure 5E and Supplementary Table 5). The collagen fiber length (CFL) appeared to increase in the anterior and posterior surfaces of both lenticules in the FEMTO LDV group and of the fresh lenticules in the VisuMax group (Figure 5F and Supplementary Table 5). Interestingly, the CFL did not change in the E-beam lenticular surfaces cut with the VisuMax (Figure 5F and Supplementary Table 5). In summary, all measured parameters of the anterior lenticular surface, the fresh and E-beam lenticules created with FEMTO LDV underwent 20.40 ± 8.20% and 16.17 ± 7.80% alterations, respectively (Figure 5G). The VisuMax affected 25.16 ± 13.81% and 28.36 ± 10.98% changes to the surface collagen morphometry (Figure 5G). On the posterior lenticular surface, the FEMTO LDV caused slightly less change to the collagen morphometry than the anterior side, where a 15.38 ± 6.30% and 15.37 ± 8.79% overall change was noted in the fresh and E-beam lenticules, respectively (Figure 5H). The VisuMax affected the collagen morphometry the most on the posterior lenticular surface, where a 23.29 ± 10.04% and 28.34 ± 9.50% change was encountered in the fresh and E-beam lenticules, respectively. In the radar maps, SMILE-derived lenticules showed the most obvious deviations from the bulk tissue (Figures 5G, H).

## 4 Discussion

The use of E-beamed cornea-derived lenticule as a possible additive tissue for stromal keratophakia, is an exciting opportunity that potentially addresses the increasing global demand for corneal grafts (36). Following our findings on the potential application of E-beam irradiated donor corneas as sources for lenticules for stromal keratophakia (19), we wanted to study the cutting consistency, cutting surface quality, and impact on the collagen morphology of a new lenticule procedure, CLEAR, compared to SMILE. It appeared that E-beam irradiated corneas could be readily shaped into lenticules with both commercially available femtosecond laser systems. However, we found several differences in the outcomes. First, the E-beamed lenticules that were cut using FEMTO LDV were significantly thicker than the fresh samples as opposed to those created with VisuMax, which had similar thickness as the fresh lenticules. Second, the surface roughness of E-beamed lenticules incised with FEMTO LDV did not differ significantly from the fresh lenticules. This contrasted with the SMILE lenticules, which showed notably smoother surfaces on the E-beamed than the fresh lenticules. Third, the FEMTO LDV induced less cumulative changes to the collagen morphology on the surfaces of fresh and E-beamed lenticules than the VisuMax.

One of the well-established advantages of femtosecond laser is the precision with which it can sculpt corneal tissue (37). In this study, we demonstrated that the FEMTO LDV system’s laser cutting accuracy was significantly impacted by the E-beam treatment in the corneas—-as opposed to VisuMax, where no significant deviation was observed. We have previously shown that E-beam treatment-induced changes in collagen type I, glycoproteins, and particularly, glycosaminoglycans (GAGs) (19). It is not known if the lenticular thickness deviation occurred proportionate to the refractive correction since all lenticules were created of the same thickness, however, optimization of the laser parameters may reduce this deviation. We used laser parameters that were consistent with our clinical practice, hence it shows the sensitivity of hydration of lenticule creation, with a low nJ-energy laser system. This is especially important with the newer lenticule procedures, e.g., CLEAR, SILK, and SmartSight that all use low nJ-energy laser systems (5).

**FIGURE 6:**
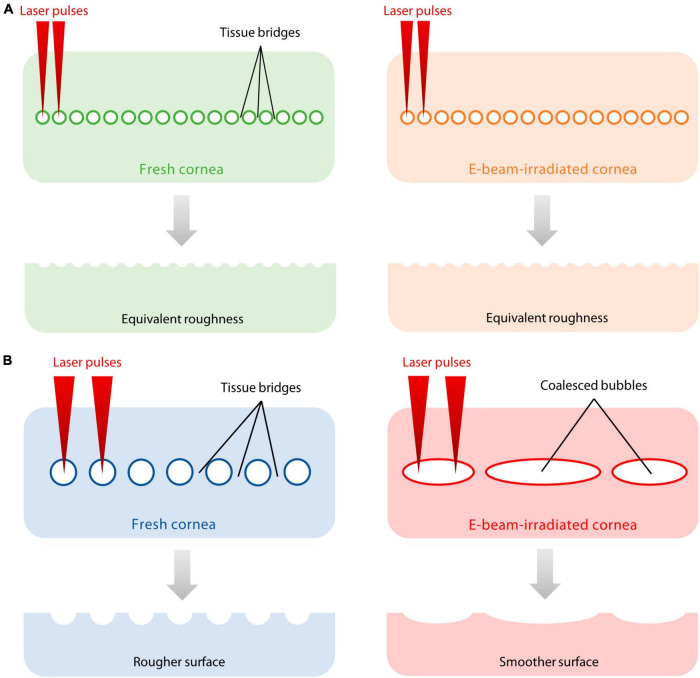
Proposed explanation of differences in surface roughness on fresh and E-beamed lenticules. **(A)** The FEMTO LDV did not rely on the generation of cavitation bubbles to cleave the stromal material. Instead, it placed laser-generated plasmas closer to each other, leaving smaller gaps between laser spots and finer tissue bridges. **(B)** The VisuMax produced a series of cavitation bubbles that created a plane of separation in the corneal stroma. Thicker tissue bridges formed between the lenticule and corneal stroma due to laser spot separation. The tendency of cavitation bubble coalescence in the E-beam-treated cornea resulted in the elimination of much of the tissue bridges, producing a smoother incisional surface compared to the fresh counterpart.

The quality of the cut stromal surface is a clinically important factor as a smoother surface is likely to improve the optical properties of a lenticular graft, early visual recovery, and visual quality (29, 31). AFM images obtained in the study, although not directly comparable due to differences in scale, demonstrated the presence of nanometre scale imperfections which was concordant with SEM images obtained. The VisuMax produced a series of cavitation bubbles that created a plane of separation in the corneal stroma. Tissue bridges formed between the lenticule and corneal stroma due to laser spot separation. In contrast, the low nJ-energy, high-frequency femtosecond laser system, such as the FEMTO LDV, does not rely on cavitation bubbles to create a dissection plane but uses the generated plasmas, which are placed closer together to cleave the stromal material (38). After lenticular extraction, the residual material from the tissue bridges contributes to the texture of the lenticular surface. Because of the minimal dependence on cavitation bubbles to separate tissue, the roughness of the lenticular surfaces cut with FEMTO LDV was consistent whether on the anterior and posterior sides and in the fresh or E-beam irradiated corneas (Figure 6A). In contrast, the surfaces of E-beamed lenticules fashioned with VisuMax were smoother than the fresh specimens, an observation in line with our previous finding (19). It was likely that the tendency of cavitation bubble coalescence in the E-beamed corneas resulted in the elimination of much of the tissue bridges (Figure 6B).

Although the femtosecond laser-created photodisruptive process purportedly reduces collateral damage to the corneal stroma, the heat and mechanical effects could create collagen fibrillar derangements at the incisional surface (34). The disturbances presented as increased reflectivity under *in vivo* confocal microscopy in previous studies and were found to attenuate over time (39, 40). The present study is the first to quantify collagen morphology derangement through multiphoton microscopy in the context of examining incisional surfaces created by femtosecond lasers. The magnitude of the collagen structure disruption appeared to be equally greater in the fresh and E-beamed lenticules in the VisuMax group compared to those in the FEMTO LDV group, however, the differences did not reach statistical significance. The non-statistical significance was likely due to the small sample size in each experimental group. However, we consistently saw the results of the representative samples trending in similar directions, albeit in different magnitudes.

It is unknown whether a difference in femtosecond laser energy level would affect the tissue surface modulus of the lenticules. In the future, the application of AFM with micro-rheology features could potentially resolve the correlation of tissue surface modulus to roughness and eventual tissue integration. Nevertheless, after implantation, despite the variations of incisional surface roughness and derangement of collagen morphology, it may not affect the patient’s vision prolonged due to continued stromal remodeling (10, 39–41). The interface between the lenticule and host stroma may heal and become unnoticeable given sufficient time (14, 39, 40, 42). The minimally invasive nature of the lenticule reimplantation procedure does not extensively disrupt the ocular surface, ensuring the minimal involvement of ocular surface antigen-presenting cells (43), hence there is minimal effect on stromal remodeling and tissue bio-integration in the long term.

For the purposes of creating lenticules in E-beam-treated corneas, using fresh donor corneas as the baseline, the VisuMax outperformed FEMTO LDV with respect to cutting consistency. However, the FEMTO LDV yielded smoother incisional surfaces and fewer surface collagen morphology changes. We concluded that both femtosecond laser systems were feasible to generate lenticules from E-beam irradiated tissues with some caveats. First, the thicker cut in FEMTO LDV-created lenticules showed the sensitivity of hydration with a low nJ-energy femtosecond laser. Nevertheless, further research is required to determine the significance of whether the thickness discrepancy can be amended with different laser settings. Second, the smoother surface and attenuated surface collagen morphology alteration in FEMTO LDV-cut lenticules could result in earlier visual recovery in patients who undergo stromal keratophakia. In the long term, the visual outcomes of either FEMTO LDV- or VisuMax-incised lenticules are likely to be the same due to the stromal remodeling and the minimally invasive nature of the stromal keratophakia.

## Data availability statement

The original contributions presented in this study are included in this article/Supplementary material, further inquiries can be directed to the corresponding authors.

## Ethics statement

Ethical approval was not required for the studies on humans in accordance with the local legislation and institutional requirements because commercially available donor corneas were used.

## Author contributions

JC: Formal analysis, Investigation, Visualization, Writing – original draft, Writing – review & editing. EH: Data curation, Formal analysis, Investigation, Methodology, Writing – review & editing. CL: Project administration, Supervision, Visualization, Writing – review & editing. AK: Conceptualization, Methodology, Project administration, Resources, Supervision, Writing – review & editing. JS: Conceptualization, Methodology, Project administration, Validation, Writing – review & editing. Y-CL: Investigation, Methodology, Visualization, Writing – review & editing. AR: Conceptualization, Funding acquisition, Investigation, Project administration, Supervision, Visualization, Writing – review & editing. JM: Conceptualization, Funding acquisition, Investigation, Methodology, Resources, Supervision, Writing – review & editing.
