# Supplementary material for: Incisional surface quality of electron-beam irradiated cornea-extracted lenticule for stromal keratophakia: high nJ-energy vs. low nJ-energy femtosecond laser
Source: Front Med (Lausanne). 2023 Dec 15;10:1289528. doi: 10.3389/fmed.2023.1289528 (PMC10754972; doi:10.3389/fmed.2023.1289528)
Supplement: Supplementary file 1 [file Data_Sheet_1.docx]

**Incisional surface quality of electron-beam irradiated cornea-extracted lenticule for stromal keratophakia: High nJ-energy vs. low nJ-energy femtosecond laser**

**Authors**: Jian S. Chan^1,2^, Evelina Han^3^, Chris H. L. Lim^1,2,4^, Arthur C. Kurz^5^, Jeremy Shuman^5^, Yu-Chi Liu^3,6,7^, Andri K. Riau^3,6*^, Jodhbir S. Mehta^3,6,7*^

**Affiliations**:

^1^Faculty of Medicine and Health, University of New South Wales, Sydney, NSW 2052, Australia

^2^Department of Ophthalmology, National University Health System, Singapore 119228, Singapore

^3^Tissue Engineering and Cell Therapy Group, Singapore Eye Research Institute, Singapore 169856, Singapore

^4^Yong Loo Lin School of Medicine, National University of Singapore, Singapore 119077, Singapore

^5^Lions World Vision Institute, Tampa, FL 33605, USA

^6^Ophthalmology and Visual Sciences Academic Clinical Programme, Duke-NUS Medical School, Singapore 169857, Singapore

^7^Singapore National Eye Centre, Singapore 168751, Singapore

***Corresponding authors**:

Dr. Andri Riau

Phone: (65) 65767488

Email: andri.kartasasmita.riau@seri.com.sg

Prof. Jodhbir Mehta

Phone: (65) 63227478

Email: jodhbir.s.mehta@singhealth.com.sg

**SUPPLEMENTARY FIGURE**

**

**

**Supplementary Figure 1.** Surface roughness profiles in the center and periphery of the corneal caps and stromal beds. Corneal caps and stromal beds were used as surrogate anterior and posterior surfaces of lenticules, respectively. (A) Root mean square values or R_q_ were similar in the center and periphery of lenticules that underwent the same pre-treatment of the corneas: fresh or E-beam irradiated, and lenticule extraction with the same femtosecond laser system: FEMTO LDV (L) or VisuMax (V). (B) Similarly, the R_z_ in the center of the caps or beds was not significantly different than that in the periphery.

**SUPPLEMENTARY TABLES**

**Supplementary Table 1.** Thickness of lenticules in situ measured with anterior segment-optical coherence tomography (AS-OCT).

| **Position (mm)** | **LDV (L)** | | | **VisuMax (V)** | | |
| --- | --- | --- | --- | --- | --- | --- |
|  | **Fresh (µm)** | **E-beam (µm)** | **p** | **Fresh (µm)** | **E-beam (µm)** | **p** |
| -3 | 22.0±7.5 | 29.7±5.7 | *0.015* | 82.0±16.0 | 76.1±13.4 | 0.589 |
| -2 | 50.0±18.0 | 75.7±9.0 | *0.004* | 100.8±14.0 | 99.9±14.5 | 0.937 |
| -1 | 72.7±16.2 | 89.0±8.2 | *0.015* | 109.7±10.6 | 106.4±9.2 | 0.589 |
| 0 (central) | 105.7±13.6 | 118.7±5.9 | *0.026* | 110.1±5.8 | 108.1±12.5 | 0.699 |
| +1 | 74.3±22.2 | 92.3±3.2 | 0.065 | 109.1±11.0 | 107.8±14.5 | 0.818 |
| +2 | 51.3±19.6 | 69.7±10.0 | *0.026* | 95.3±13.8 | 94.7±5.1 | 0.699 |
| +3 | 26.0±7.0 | 35.3±1.5 | *0.004* | 80.0±10.5 | 75.4±12.0 | 0.699 |

**Supplementary Table 2.** Comparison of lenticular surface roughness profile at the center and periphery measured on corneal caps and beds with atomic force microscopy (AFM).

|  | **Anterior surface** | | | | | **Posterior surface** | | | | |
| --- | --- | --- | --- | --- | --- | --- | --- | --- | --- | --- |
|  | **Centre** | | **Periphery** | | **p** | **Centre** | | **Periphery** | | **p** |
| **R_q_ (nm)** |  |  | |  | |  |  | |  | |
| Fresh (L) | 87.2±27.5 | | 89.8±35.3 | | 0.884 | 88.7±18.5 | | 70.2±12.7 | | 0.237 |
| E-beam (L) | 90.1±26.7 | | 102.2±27.0 | | 0.502 | 74.0±20.2 | | 86.87±19.8 | | 0.501 |
| Fresh (V) | 162.3±33.2 | | 175.0±49.4 | | 0.699 | 233.7±31.6 | | 193.1±22.1 | | 0.120 |
| E-beam (V) | 79.6±28.2 | | 89.3±20.6 | | 0.532 | 148.9±35.4 | | 153.1±26.7 | | 0.843 |
| **R_z_ (µm)** |  |  | |  | |  |  | |  | |
| Fresh (L) | 0.58±0.12 | | 0.61±0.18 | | 0.791 | 0.68±0.16 | | 0.66±0.16 | | 0.888 |
| E-beam (L) | 0.64±0.18 | | 0.70±0.18 | | 0.749 | 0.54±0.13 | | 0.62±0.18 | | 0.521 |
| Fresh (V) | 1.11±0.19 | | 1.17±0.44 | | 0.774 | 1.33±0.18 | | 1.26±0.27 | | 0.663 |
| E-beam (V) | 0.89±0.30 | | 0.78±0.37 | | 0.447 | 1.15±0.21 | | 1.00±0.30 | | 0.413 |

L = LDV

V = VisuMax

**Supplementary Table 3.** Lenticular surface roughness profile measured on corneal caps and beds with AFM.

|  | **Anterior surface** | | | | | **Posterior surface** | | | | |
| --- | --- | --- | --- | --- | --- | --- | --- | --- | --- | --- |
|  | **Fresh** | **E-beam** | | **p (Fresh vs. E-beam)** | | **Fresh** | **E-beam** | | **p (Fresh vs. E-beam)** | |
| **LDV (L)** |  | |  | |  |  | |  | |  |
| R_q_ (nm) | 88.5±29.8 | 96.1±25.6 | | 0.967 | | 79.4±15.9 | 80.5±19.2 | | 0.974 | |
| R_z_ (µm) | 0.60±0.14 | 0.67±0.17 | | 0.872 | | 0.67±0.15 | 0.58±0.15 | | 0.694 | |
| **VisuMax (V)** |  | |  | |  |  | |  | |  |
| R_q_ (nm) | 168.7±39.8 | 84.5±23.5 | | *0.001* | | 213.4±28.2 | 151.0±29.6 | | *0.005* | |
| R_z_ (µm) | 1.14±0.32 | 1.29±0.22 | | 0.305 | | 0.84±0.32 | 1.07±0.25 | | 0.847 | |
| **p of R_q_ (L vs. V)** | *0.003* | 0.853 | |  | | *1.27x10^-9^* | *0.001* | |  | |
| **p of R_z_ (L vs. V)** | *0.007* | 0.690 | |  | | *1.97x10^-4^* | 0.510 | |  | |

L = LDV

V = VisuMax

**Supplementary Table 4.** Collagen morphometry of fresh and E-beamed bulk stroma analyzed with multiphoton microscopy.

|  | **Fresh** | **E-beam** | **p** |
| --- | --- | --- | --- |
| CART | 67.08±8.94 | 69.94±6.09 | 0.999 |
| CFD | 43.18±2.56 | 46.21±2.86 | 0.485 |
| CARD | 7.98±0.46 | 7.88±0.72 | 0.937 |
| CFT (µm) | 3.18±0.25 | 3.21±0.27 | 0.818 |
| CFL (µm) | 12.19±1.13 | 12.47±47 | 0.999 |

CART = collagen area ratio in tissue

CFD = collagen fiber density

CARD = collagen area reticulation density

CFT = collagen fiber thickness

CFL = collagen fiber length

**Supplementary Table 5.** Collagen morphometry of lenticular surfaces relative to the bulk stroma analyzed with multiphoton microscopy.

|  | **Anterior surface** | | | **Posterior surface** | | |
| --- | --- | --- | --- | --- | --- | --- |
|  | **Fresh** | **E-beam** | **p (Fresh vs. E-beam)** | **Fresh** | **E-beam** | **p (Fresh vs. E-beam)** |
| **LDV (L)** |  |  |  |  |  |  |
| CART | 0.85±0.17 | 0.88±0.06 | 0.998 | 0.82±0.04 | 0.91±0.05 | 0.600 |
| CFD | 1.07±0.06 | 0.97±0.08 | 0.816 | 0.97±0.04 | 0.95±0.08 | 0.998 |
| CARD | 1.10±0.07 | 0.98±0.06 | 0.652 | 1.05±0.09 | 1.00±0.02 | 0.918 |
| CFT | 0.80±0.07 | 0.82±0.02 | 0.991 | 0.80±0.05 | 0.83±0.02 | 0.940 |
| CFL | 1.24±0.21 | 1.33±0.12 | 0.974 | 1.22±0.10 | 1.39±0.09 | 0.652 |
| **VisuMax (V)** |  |  |  |  |  |  |
| CART | 0.77±0.11 | 0.55±0.04 | 0.387 | 0.72±0.06 | 0.68±0.03 | 0.940 |
| CFD | 1.05±0.07 | 1.16±0.04 | 0.510 | 1.04±0.05 | 0.96±0.11 | 0.894 |
| CARD | 1.20±0.10 | 1.37±0.18 | 0.985 | 1.20±0.11 | 1.38±0.20 | 0.852 |
| CFT | 0.70±0.04 | 0.67±0.07 | 0.972 | 0.71±0.05 | 0.68±0.06 | 0.983 |
| CFL | 1.44±0.28 | 1.01±0.08 | 0.557 | 1.32±0.17 | 1.23±0.07 | 0.947 |
| **p of CART*** | 0.978 | 0.050 |  | 0.623 | 0.095 |  |
| **p of CFD*** | 0.999 | 0.333 |  | 0.706 | 0.999 |  |
| **p of CARD*** | 0.855 | 0.379 |  | 0.765 | 0.427 |  |
| **p of CFT*** | 0.673 | 0.371 |  | 0.603 | 0.255 |  |
| **p of CFL*** | 0.933 | 0.263 |  | 0.947 | 0.590 |  |

*indicates comparison between L and V groups.

CART = collagen area ratio in tissue

CFD = collagen fiber density

CARD = collagen area reticulation density

CFT = collagen fiber thickness

CFL = collagen fiber length

**SUPPLEMENTARY VIDEOS**

**Supplementary Video 1.** Three-dimensional AS-OCT of the fresh cornea after Cornea Lenticule Extraction for Advanced Refractive-Correction (CLEAR) lenticule incision with FEMTO LDV. AS-OCT scan was taken before the lenticule was extracted.

**Supplementary Video 2.** Three-dimensional AS-OCT of the E-beam-irradiated cornea after CLEAR lenticule incision with FEMTO LDV. AS-OCT scan was taken before the lenticule was extracted.

**Supplementary Video 3.** Three-dimensional AS-OCT of the fresh cornea after Small Incision Lenticule Extraction (SMILE) lenticule incision with VisuMax. AS-OCT scan was taken before the lenticule was extracted.

**Supplementary Video 4.** Three-dimensional AS-OCT of the E-beam-irradiated cornea after SMILE lenticule incision with VisuMax. AS-OCT scan was taken before the lenticule was extracted.
